# Cornual heterotopic pregnancy: a case report

**DOI:** 10.4076/1752-1947-3-7233

**Published:** 2009-06-23

**Authors:** Olivier Poujade, Guillaume Ducarme, Dominique Luton

**Affiliations:** 1 Department of Gynecology and Obstetrics, Hôpital Beaujon, Bld du Général Leclerc, 92110 Clichy, France

## Abstract

**Introduction:**

Cornual heterotopic pregnancy is a very rare condition; its incidence remains unknown. We report a case of cornual heterotopic pregnancy managed by laparoscopy and guided methotrexate injection into the cornual sac.

**Case presentation:**

A cornual heterotopic pregnancy was diagnosed at 9 weeks of amenorrhoea in a 31-year-old healthy woman. Ultrasound examination showed a well-formed intrauterine gestation without detectable fetal heart pulsation, together with a gestational sac situated in the right cornual region. After uterine evacuation under ultrasound guidance, the diagnosis of cornual pregnancy was confirmed on laparoscopy followed by methotrexate injection into the cornual gestational sac.

**Conclusions:**

Cornual heterotopic pregnancy is a very rare and potentially dangerous condition. Diagnosis of cornual pregnancy could be made on ultrasound examination in this patient. Laparoscopy was useful as an alternative in confirming the diagnosis and aided further treatment.

## Introduction

Heterotopic pregnancy is defined as the coexistence of intrauterine pregnancy and ectopic pregnancy. The incidence of heterotopic pregnancy is estimated to be 1/30,000 in spontaneous pregnancy but much higher than 1/3600 to 1/100 when associated with *in vitro* fertilization (IVF) according to more recent literature. It is probably in the range of 1% to 3% [1,2].

Cornual pregnancy occurs at the funnel-shaped area in the upper uterine body that receives the insertion of the Fallopian tubes [3], the cornual region of the uterus. Its occurrence rate ranges from 1/2500 to 1/5000 live births and represents 1% of ectopic pregnancies [4].

The incidence of heterotopic pregnancy with extrauterine gestation located in the cornual area is not known, nevertheless, the incidence of cornual heterotopic pregnancy is estimated to be 1/3600 IVF pregnancies [5]. We present one case of spontaneous cornual heterotopic pregnancy diagnosed by ultrasound, and managed by laparoscopic-guided methotrexate injection into the cornual sac.

## Case presentation

A healthy 31-year-old woman presented at 9 weeks of amenorrhoea with spotting and lower abdominal pain. She had a previous history of four term pregnancies and one spontaneous abortion, and her current pregnancy occurred spontaneously. Her vital signs were stable, and physical examination revealed a slight pain in the right lower quadrant of the abdomen without rebound. Serum beta-human chorionic gonadotropin (beta-HCG) level was 12,237 IU/litre and haemoglobin level was 10.9 g/dl.

**Figure 1:**
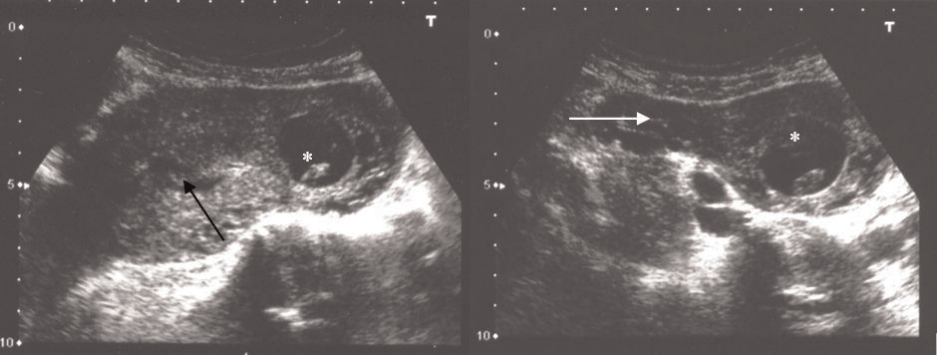
**Transvaginal ultrasound of the uterus (transverse section) before evacuation of the non-viable intrauterine pregnancy.** The image in the left panel shows an intrauterine gestation (black arrow) coexisting with an ectopic cornual pregnancy (*) with a sac of 25 mm in diameter, containing an embryo with a crown rump length of 13 mm. The image in the right panel shows the ectopic pregnancy (*) located in the right cornual region in continuity with the uterine cavity, in a funnel-shaped area in the upper uterine body that receives the insertion of the right Fallopian tube (white arrow).

A transabdominal and transvaginal ultrasound examination demonstrated a normal-looking intrauterine gestation with a sac of 35 mm in diameter and a crown-rump length (CRL) of 27 mm without positive fetal heart rate, consistent with a fetal age of approximately 9 weeks and 5 days of amenorrhoea. Another gestational sac was situated in the right cornual region in continuity with the uterine cavity, suggesting a cornual heterotopic pregnancy. This sac was 25 mm in diameter, containing an embryo with a CRL of 13 mm without heart cardiac activity (Figure 1). No free fluid was noticed in the cul-de-sac.

Based on ultrasound findings, the diagnosis of cornual heterotopic pregnancy was made. The patient was apprised of the diagnosis, treatment options were discussed, and after obtaining informed consent she underwent a uterine evacuation and a laparoscopy under general anaesthesia.

**Figure 2:**
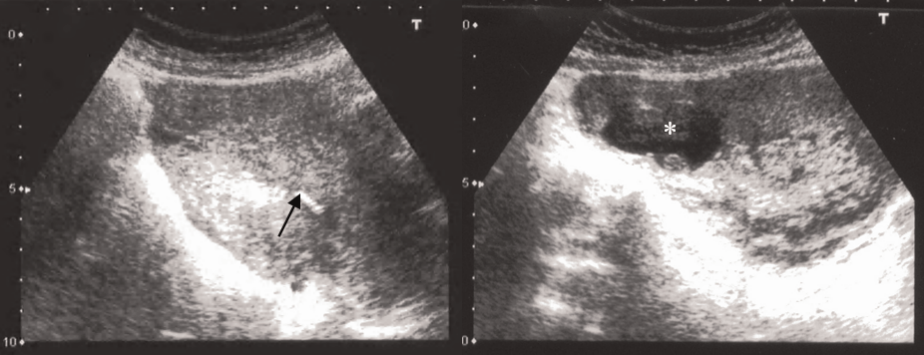
**Transabdominal ultrasound of the uterus after evacuation of the non-viable intrauterine pregnancy.** The image in the left panel is a sagittal section showing no retained products of conception after the uterine evacuation (black arrow). The image in the right panel is a transverse section showing the persistence of the right cornual gestational sac (*) in continuity with the uterine cavity.

**Figure 3:**
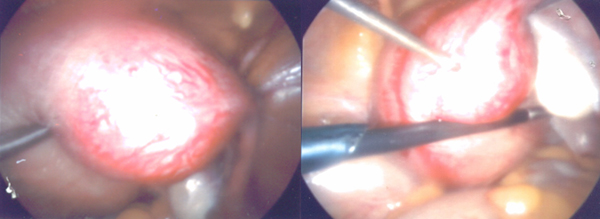
**Laparoscopic visualization of the cornual pregnancy and the methotrexate injection.** The image in the left panel shows the right cornual pregnancy located behind the round ligament, occurring at the funnel-shaped area in the upper uterine body, without reaching the Fallopian tube. The image in the right panel shows the methotrexate injection into the cornual sac.

The management consisted firstly of evacuation of the non-viable intrauterine pregnancy under transabdominal sonographic guidance aiming to avoid a uterine perforation (Figure 2). Secondly, a laparoscopy confirmed the diagnosis showing an enlarged uterus and a 3 cm diameter right cornual pregnancy. Both the tubes and ovaries appeared normal, no cornual rupture had occurred and a minimal haemoperitoneum was present. A laparoscopic-guided methotrexate (50 mg/m²) injection was performed into the cornual sac (Figure 3).

The patient recovered uneventfully and was discharged from the hospital within 48 hours. She was followed up weekly, including clinical examination, transvaginal ultrasound examination and plasmatic beta-HCG level. Her follow-up beta-HCG was 6705 IU/litre 7 days after, dropped to 3884 IU/litre 1 week later, continued to fall as expected, and dropped to normal 2 months later. The cornual pregnancy finally disappeared under ultrasound examination 2 months later.

## Discussion

Cornual heterotopic pregnancy is very rare, although its prevalence has probably increased due to the emergence of assisted reproductive technologies. Thirty-two cases have been published since 1990, whereas only nine cases were reported before the last decade.

Diagnosis of this condition is difficult due to the existence of the intrauterine gestational sac. The most frequent danger lies in the non-recognition of the condition and subsequent uterine rupture at a more advanced gestation. Cornual rupture in a context of cornual heterotopic pregnancy occurs in approximately 48.6% of cases [5], and usually results in brisk haemorrhage due to the fact that the gestational sac lies next to an extensive vascular area and the uterine artery. Maternal mortality is estimated to occur in 2% to 2.5% of cases [4].

The treatment options include medical or surgical management. Expectant management does not seem adequate, since the risk of rupture is considerable. Medical management is recommended in the case of a symptom-free patient with a Beta-HCG level lower than 5000 IU/litre, and principally concerns the use of methotrexate (systemic injection, ultrasound-guided injection to the cornual pregnancy, or hysteroscopic injection). Laparoscopic delivery of methotrexate was chosen in this case, in preference to an ultrasound guided procedure, aiming to confirm the diagnosis of cornual pregnancy and to avoid the risk of bleeding or even rupture at the site of the needle puncture [6]. In the case of a viable intrauterine pregnancy and methotrexate contraindication, injection of potassium chloride under transvaginal ultrasound or under a laparoscopy procedure appears to be the most adequate option. Patients with failure of medical treatment would require secondary surgery.

From the surgical point of view, laparoscopic procedures are more common than laparotomy, although conversion to laparotomy occurs in approximately 27% of cases due to haemoperitoneum or technical difficulties. The different laparoscopic options are resection of cornua, Vicryl loop placement, and methotrexate and/or potassium chloride injection into the amniotic sac.

When performing these procedures two risks have to be taken into account, firstly wedge resection and complete extraction of the pregnancy increase the risk of a large amount of blood loss during the procedure, and the potential risk of hysterectomy. Secondly, cornual resection may weaken the uterine musculature, increasing the risk of rupture during a subsequent pregnancy. It may, however, negate the complications of medically treated cornual pregnancy including the need for serial follow-up and the risks of delayed haemorrhage or rupture.

Habana *et al.*[5] studied the outcomes of women undergoing surgery versus medical treatment, and demonstrated the benefits of surgery in terms of miscarriage (13% versus 50%, p < 0.05) and live birth rate (60.9% versus 50%).

The incidence of recurrent cornual ectopic pregnancies is unknown; nevertheless, this finding has already been reported [7,8]. As suggested by van der Weiden and Karsdorp [7], assisted reproductive techniques and conservative methods of management may increase the incidence of recurrence.

## Conclusions

Cornual pregnancy remains a potentially dangerous condition. Laparoscopy appears to be safe and effective, allowing a reliable and early diagnosis, and should be recommended in the absence of cornual rupture signs. In the case of rupture, cornual resection under laparotomy remains the preferred method.

## Abbreviations

Beta-HCG: beta-human chorionic gonadotrophin; CRL: crown-rump length; IVF: *in vitro* fertilization.

## Consent

Written informed consent was obtained from the patient for publication of this case report and any accompanying images. A copy of the written consent is available for review by the Editor-in-Chief of this journal.

## Competing interests

The authors declare that they have no competing interests.

## Authors' contributions

OP performed the laparoscopy and contributed to the writing of the manuscript. GD and DL contributed to the writing of the manuscript. GD has made substantial contributions to conception and analysis of the data. DL has given final approval of the version to be published. All authors read and approved the final manuscript.
